# Supplementary material for: Gorlin Syndrome: Recent Advances in Genetic Testing and Molecular and Cellular Biological Research
Source: Int J Mol Sci. 2020 Oct 13;21(20):7559. doi: 10.3390/ijms21207559 (PMC7590212; doi:10.3390/ijms21207559)
Supplement: Supplementary file 1 [file ijms-21-07559-s001.pdf]

Table S1: The list of the mutations in patients with GS.

PTCH1 mutation of Gorlin syndrome

Mutation type	Nucleotide change	Amino acid changes	Exon/intron no.	Characterization	Reference no.
Missense	c.317T>G	p.Leu106Arg	Exon2	Germline	[1]
Missense	c.328G>T	p.Gly110Trp	Exon2	Germline	[2]
Missense	c.591G>C	p.Trp197Cys	Exon4	Germline	[3]
Missense	c.688A>C	p.Thr230Pro	Exon5	Germline	[4]
Missense	c.689C>G	p.Thr230Arg	Exon5	.	[3]
Missense	c.709G>A	p.Glu237Lys	Exon5	Germline	[5]
Missense	c.863G>A	p.Gly288Asp	Exon6	Germline	[6]
Missense	c.890T>C	p.Leu297Pro	Exon6	.	[3]
Missense	c.1115T>G	p.Met372Arg	Exon8	.	[7]
Missense	c.1162A>T	p.Asn388Tyr	Exon8	Germline	[8]
Missense	c.1247C>G	p.Thr416Ser	Exon9	Somatic	[6]
Missense	c.1325T>A	p.Val442Glu	Exon9	Germline	[9]
Missense	c.1346T>A	p.Met449Lys	Exon9	Germline	[10]
Missense	c.1415C>A	p.Ala472Asp	Exon10	Germline	[11]
Missense	c.1436T>G	p.Leu479Arg	Exon10	Germline	[12]
Missense	c.1439C>T	p.Ser480Leu	Exon10	.	[3]
Missense	c.1450G>A	p.Gly484Arg	Exon10	.	[3]
Missense	c.1451G>T	p.Gly484Val	Exon10	Germline	[11]
Missense	c.1450G>A	p.Gly484Arg	Exon10	Germline	[13]
Missense	c.1511C>T	p.Pro504Leu	Exon11	Germline	[13]
Missense	c.1517T>G	p.Leu505Arg	Exon11	Germline	[14]
Missense	c.1525G>C	p.Gln509Arg	Exon11	Germline	[15][16]
Missense	c.1526G>T	p.Gly509Val	Exon11	Germline	[12]
Missense	c.1526G>A	p.Gly509Asp	Exon11	Germline	[3][17]
Missense	c.1555G>C	p.Ala519Pro	Exon11	.	[3]
Missense	c.1660A>C	p.Ser554Arg	Exon12	Germline	[2]
Missense	c.1712G>C	p.Arg571Pro	Exon12	.	[3]
Missense	c.1939A>T	p.Ser647Cys	Exon14	Germline	[1]
Missense	c.1941C>A	p.Ser647Arg	Exon14	Germline	[13]
Missense	c.2183C>T	p.Thr728Met	Exon14	.	[3]
Missense	c.2186A>T	p.Lys729Met	Exon14	Germline	[18][19][20]
Missense	c.2250G>C	p.Lys750Asn	Exon14	.	[3]
Missense	c.2414T>G	p.Ile805Arg	Exon15	.	[3]
Missense	c.2447A>G	p.Gln816Arg	Exon15	.	[3]
Missense	c.2465T>C	p.Leu822Pro	Exon15	Germline	[13]
Missense	c.2776T>C	p.Trp926Arg	Exon17	Germline	[13]
Missense	c.2917C>A	p.Gln973Lys	Exon18	.	[3]
Missense	c.3062A>G	p.Tyr1021Cys	Exon18	Germline	[21]

Missense	c.3146A>T	p.Asn1049Ile	Exon18	Germline	[22]
Missense	c.3277G>C	p.Glu1093Arg	Exon19	Germline	[19],[23]
Missense	c.3394T>C	p.Ser1132Pro	Exon20	Germline	[24]
Missense	c.3395C>A	p.Ser1132Tyr	Exon20	Germline	[16]
Missense	c.3398C>T	p.Thr1133Ile	Exon20	Germline	[25]
Missense	c.3440T>G	p.Phe1147Cys	Exon20	Germline	[6]
Missense	c.3499G>A	p.Gly1167Arg	Exon21	Germline	[6],[12],[26]
Missense	c.3956G>A	p.Arg1319His	Exon23	Germline	[11]
Missense	c.4314G>T	p.Glu1438Asp	Exon23	Germline	[27]
Nonsense	c.205A>T	p.Lys69*	Exon2	Germline	[3]
Nonsense	c.233G>A	p.Trp78*	Exon2	Germline	[28]
Nonsense	c.250C>T	p.Gln84*	Exon2	Germline	[4]
Nonsense	c.279C>A	p.Tyr93*	Exon2	Germline	[29]
Nonsense	c.294C>A	p.Cys98*	Exon2	Germline	[3]
Nonsense	c.305T>A	p.Leu102*	Exon2	Germline	[4]
Nonsense	c.343G>T	p.Gly115*	.	.	Unpublished
Nonsense	c.379G>T	p.Glu127*	Exon2	.	[30]
Nonsense	c.387G>A	p.Trp129*	Exon2	Germline	[31]
Nonsense	c.403C>T	p.Arg135*	Exon3	Somatic	[32],[33]
Nonsense	c.403C>T	p.Arg135*	Exon3	Germline	[34]
Nonsense	c.466C>T	p.Gln156*	Exon3	Germline	[3]
Nonsense	c.478C>T	p.Gln160*	Exon3	Germline	[35]
Nonsense	c.550C>T	p.Gln184*	Exon3	Germline	[36]
Nonsense	c.661G>T	p.Glu221*	Exon5	.	[30]
Nonsense	c.666T>A	p.Tyr222*	.	.	Unpublished
Nonsense	c.672C>A	p.Tyr224*	Exon5	.	[13]
Nonsense	c.707G>A	p.Trp236*	Exon5	.	[3]
Nonsense	c.722T>G	p.Leu241*	.	.	[37]
Nonsense	c.811del	p.Ile271*	.	.	[38]
Nonsense	c.820C>T	p.Gln274*	Exon6	.	[30]
Nonsense	c.833G>A	p.Trp278*	Exon6	Germline	[11]
Nonsense	c.873C>G	p.Tyr291*	Exon6	Germline	[28]
Nonsense	c.938C>G	p.Ser313*	Exon6	.	[30]
Nonsense	c.1012C>T	p.Gln338*	Exon7	Somatic	[32][33]
Nonsense	c.1018G>T	p.Glu340*	Exon7	Germline	[39]
Nonsense	c.1081C>T	p.Gln361*	Exon8	.	[3]
Nonsense	c.1093C>T	p.Gln365*	Exon8	Germline	[40]
Nonsense	c.1119C>G	p.Tyr373*	.	.	[3]
Nonsense	c.1138G>T	p.Glu380*	Exon8	Germline	[12]
Nonsense	c.1148C>G	p.Ser383*	Exon8	Germline	[41]
Nonsense	c.1160G>A	p.Trp387*	.	.	[42]
Nonsense	c.1196G>A	p.Trp399*	Exon8	Germline	[43]

Nonsense	c.1198C>T	p.Gln400*	Exon8	.	[3]
Nonsense	c.1237C>T	p.Gln413*	Exon9	Germline	[19]
Nonsense	c.1380G>A	p.Trp460*	Exon10	.	[3]
Nonsense	c.1585A>T	p.Lys529*	Exon11	Germline	[38]
Nonsense	c.1691T>G	p.Leu564*	Exon12	.	[3]
Nonsense	c.1712G>C	p.Arg571Pro	Exon12	.	[3]
Nonsense	c.1804C>T	p.Arg602*	Exon13	.	[3]
Nonsense	c.1810G>T	p.Glu604*	Exon13	.	[44]
Nonsense	c.1975C>T	p.Gln659*	Exon14	.	[3]
Nonsense	c.1987C>T	p.Gln663*	Exon14	Germline	[19][38]
Nonsense	c.2062C>T	p.Gln688*	Exon14	Germline	[19][34]
Nonsense	c.2098C>T	p.Gln700*	Exon14	.	[3]
Nonsense	c.2170G>T	p.Glu724*	Exon14	.	[3]
Nonsense	c.2308C>T	p.Arg770*	Exon15	.	[3]
Nonsense	c.2359G>T	p.Glu787*	Exon15	.	[3]
Nonsense	c.2391C>A	p.Tyr787*	Exon15	Germline	[45]
Nonsense	c.2446C>T	p.Gln816*	Exon15	.	[3]
Nonsense	c.2557C>T	p.Gln853*	Exon15	.	[3]
Nonsense	c.2619C>A	p.Tyr873*	Exon16	Germline	[1][6][13][46]
Nonsense	c.2760C>A	p.Tyr920*	Exon17	Germline	[26][47]
Nonsense	c.2833C>T	p.Arg945*	Exon17	Germline	[48]
Nonsense	c.2908G>T	p.Glu970*	Exon18	Germline	[2]
Nonsense	c.3027C>A	p.Tyr1009*	Exon18	.	[3]
Nonsense	c.3027C>G	p.Tyr1009*	Exon18	.	[3]
Nonsense	c.3053G>A	p.Trp1018*	Exon18	.	[49]
Nonsense	c.3058C>T	p.Gln1020*	Exon18	Germline	[3][47]
Nonsense	c.3062A>G	p.Tyr1021Cys	Exon18	.	[21]
Nonsense	c.3265T>C	p.Ser1089Pro	.	.	[35]
Nonsense	c.3277G>T	p.Gly1093*	Exon19	.	[30]
Nonsense	c.3422C>T	p.Ala1141Val	.	.	[50]
Small out-of-frame deletions	c.220delA	p.Ala75Argfs*5	Exon2	Germline	[4]
Small out-of-frame deletions	c.239_240delGA	p.Arg80Serfs*9	Exon2	Germline	[43]
Small out-of-frame deletions	c.256_257delCT	p.Leu87Ilefs*2	Exon2	Germline	[34]
Small out-of-frame deletions	c.265_287del23	p.Gly91Argfs*41	Exon2	Germline	[16]
Small out-of-frame deletions	c.278delA	p.Tyr93Serfs*24	Exon2	Germline	[51]
Small out-of-frame deletions	c.331delG	p.Ala111Profs*6	Exon2	Germline	[1]
Small out-of-frame deletions	c.479_482delAGAC	p.Gln160Profs*10	Exon3	Germline	[52]
Small out-of-frame deletions	c.747_759del	p.Gly250Cysfs*15	.	.	Unpublished
Small out-of-frame deletions	c.768_777del10	p.Asn258Leufs*8	Exon6	Germline	[35]
Small out-of-frame deletions	c.808delA	p.Ile271*	Exon6	Germline	[38]
Small out-of-frame deletions	c.865delC	p.His289Metfs*35	Exon6	Germline	[2]
Small out-of-frame deletions	c.912delC	p.Ala306Profs*18	Exon6	Germline	[17]

Small out-of-frame deletions	c.941delC	p.Lys315Asnfs*9	Exon6	Germline	[34]
Small out-of-frame deletions	c.980delG	p.Cys327Phefs*5	Exon7	Germline	[47]
Small out-of-frame deletions	c.1042_1043delAA	p.Lys348Glufs*88	.	.	[50]
Small out-of-frame deletions	c.1208_1209delAT	p.Tyr403Cysfs*33	Exon8	Germline	[13]
Small out-of-frame deletions	c.1291delC	p.Leu431*	Exon9	Germline	[13]
Small out-of-frame deletions	c.1341_1344delACTC	p.Leu446fs*	Exon9	.	[14]
Small out-of-frame deletions	c.1383delC	p.Asp460fs*	Exon10	.	[14]
Small out-of-frame deletions	c.1472delT	p.Ile491Thrfs*51	Exon10	Germline	[53]
Small out-of-frame deletions	c.1615delG	p.Glu539Serfs*3	Exon12	Germline	[4]
Small out-of-frame deletions	c.1670_1671delCA	p.Thr557Serfs*69	Exon12	Germline	[43]
Small out-of-frame deletions	c.2029delA	p.Thr667Profs*16	Exon14	Germline	[43]
Small out-of-frame deletions	c.2011delC	p.His671Thrfs*22	Exon14	Germline	[17]
Small out-of-frame deletions	c.2178delC	p.Cys727Valfs*19	Exon14	Germline	[54]
Small out-of-frame deletions	c.2179delT	p.Cys727Valfs*19	Exon14	Germline	[32][33]
Small out-of-frame deletions	c.2195_2196delTC	p.Ser733Ilefs*4	Exon14	Germline	[34]
Small out-of-frame deletions	c.2196_2197delCT	p.Ser733Ilefs*4	Exon14	Germline	[6]
Small out-of-frame deletions	c.2197_2198delTC	p.Ser733Ilefs*4	Exon14	Germline	[54]
Small out-of-frame deletions	c.2287delG	p.Val763Serfs*9	Exon15	Germline	[4]
Small out-of-frame deletions	c.2395delT	p.Ser799Leufs*7	Exon15	Germline	[29]
Small out-of-frame deletions	c.2442_2452del11	p.Ile815Thrfs*10	Exon15	Germline	[55]
Small out-of-frame deletions	c.2577_2583del7	p.Phe859Leufs*42	Exon16	Germline	[12]
Small out-of-frame deletions	c.2576_2582del7	p.Phe859Leufs*42	Exon16	Germline	[4]
Small out-of-frame deletions	c.2582delG	p.Ser861Metfs*14	Exon16	Germline	[56]
Small out-of-frame deletions	c.2595delC	p.Ile868Serfs*35	Exon16	Germline	[34]
Small out-of-frame deletions	c.2719delC	p.Leu907Trpfs*17	Exon17	Germline	[34]
Small out-of-frame deletions	c.2798delC	p.Ala933Glyfs*29	Exon17	Germline	[11]
Small out-of-frame deletions	c.2824delC	p.Arg942Glyfs*20	Exon17	Germline	[32][33]
Small out-of-frame deletions	c.2833_2843del	p.Arg945Glyfs*10	Exon17	.	[3]
Small out-of-frame deletions	c.2869delC	p.Pro957Leufs*5	Exon17	Germline	[57]
Small out-of-frame deletions	c.3000_3007del8	p.Asn1000Lysfs*142	Exon18	Germline	[27]
Small out-of-frame deletions	c.3016delC	p.Leu1006Cysfs*43	Exon18	Germline	[2]
Small out-of-frame deletions	c.3042delC	p.Phe1015Serfs*34	Exon18	Germline	[13]
Small out-of-frame deletions	c.3375delC	p.Val1126Serfs*13	Exon20	Germline	[4]
Small out-of-frame deletions	c.3475delC	p.Leu1159Serfs*32	Exon21	Germline	[58]
Small out-of-frame deletions	c.3570delG	p.Leu1190*	Exon22	Germline	[27]
Small in-frame deletions	c.114del	p.Leu39Cysfs*41	Exon1	Germline	[3]
Small in-frame deletions	c.254_255del	p.Arg85Thrfs*4	Exon2	Germline	[3]
Small in-frame deletions	c.258_259del	p.Leu87Ilefs*2	Exon2	.	[3]
Small in-frame deletions	c.262_265del	p.Phe88Asnfs*28	Exon2	Germline	[30]
Small in-frame deletions	c.262_266del	p.Phe88Thrfs*50	Exon2	Germline	[3]
Small in-frame deletions	c.290del	p.Asn97Thrfs*20	.	Germline	[51]
Small in-frame deletions	c.331del	p.Ala111Profs*6	.	Germline	[30]

Small in-frame deletions	c.459del	p.Phe153Leufs*6	.	Germline	[59]
Small in-frame deletions	c.479_482del	p.Gln160Profs*10	Exon3	.	[3]
Small in-frame deletions	c.724del	p.Gln242Serfs*8	Exon5	.	[3]
Small in-frame deletions	c.771_780del	p.Asn258Leufs*8	.	.	[35]
Small in-frame deletions	c.842del	p.Met281Serfs*2	Exon6	.	[3]
Small in-frame deletions	c.865del	p.His289Metfs*35	Exon6	Germline	[2]
Small in-frame deletions	c.874del	p.Met292Trpfs*32	.	.	[30]
Small in-frame deletions	c.903del	p.Pro302Glnfs*22	.	.	[60]
Small in-frame deletions	c.922del	p.Ala308Profs*16	.	.	[49]
Small in-frame deletions	c.942del	p.Lys315Asnfs*9	.	.	[34]
Small in-frame deletions	c.1026del	p.Ile342Metfs*25	Exon7	.	[30]
Small in-frame deletions	c.1040_1049del	p.Val347Alafs*17	Exon7	.	[3]
Small in-frame deletions	c.1114del	p.Met372Cysfs*60	Exon8	.	[3]
Small in-frame deletions	c.1208_1209del	p.Tyr403Cysfs*33	Exon8	.	[13]
Small in-frame deletions	c.1279del	p.Leu427Trpfs*5	Exon9	.	[3]
Small in-frame deletions	c.1309_1329del	p.Val437_Ala443del	Exon9	.	[30]
Small in-frame deletions	c.1340_1343del	p.Leu447Serfs*8	Exon9	.	[30]
Small in-frame deletions	c.1342_1345del	p.Leu448Cysfs*7	Exon9	Germline	[28]
Small in-frame deletions	c.1348_1350del	p.Leu450del	Exon10	.	[3]
Small in-frame deletions	c.1415_1429del	p.Ala472_Leu476del	Exon10	.	[3]
Small in-frame deletions	c.1537_1539delGATor c.1540_1542delGAT	p.Asp513del or p.Asp514del	Exon11	Germline	[61]
Small in-frame deletions	c.1767_1769del	p.Leu590del	Exon13	.	[3]
Small in-frame deletions	c.1852del	p.Cys618Alafs*5	Exon14	.	[3]
Small in-frame deletions	c.(2250+1_2251-1)_(2703+1_2704-1)del	p.Val751_Gln901del	.	.	[3]
Small in-frame deletions	c.2444_2461del18	p.Ile815_Asp821delinsAsn	Exon15	Germline	[62]
Small in-frame deletions	c.2446_2448delCAG	p.Gln816del	Exon15	Germline	[16]
Small in-frame deletions	c.2612_2615del	p.Asn871Ilefs*31	Exon16	.	[3]
Small in-frame deletions	c.2748del	p.Ser917Alafs*7	Exon17	.	[3]
Small in-frame deletions	c.2793del	p.Val932Serfs*30	Exon17	.	[3]
Small in-frame deletions	c.2833_2843del	p.Arg945Glyfs*10	Exon17	.	[3]
Small in-frame deletions	c.2924del	p.Pro975Leufs*20	Exon17	.	[30]
Small in-frame deletions	c.2993del	p.Cys998Serfs*51	.	.	[50]
Small in-frame deletions	c.3000_3005del6	p.Asn1000_Thr1002delinsLys	Exon18	Germline	[12]
Small in-frame deletions	c.3042del	p.Phe1015Serfs*34	.	.	[13]
Small in-frame deletions	c.3050del	p.Phe1017Serfs*32	Exon18	.	[3]
Small in-frame deletions	c.3051_3052del	p.Phe1017Leufs*127	.	.	[12]
Small in-frame deletions	c.3055del	p.Glu1019Serfs*30	Exon18	.	[63]
Small in-frame deletions	c.3056_3059del	p.Glu1019Glyfs*29	Exon18	.	[3]
Small in-frame deletions	c.3107_3108del	p.Leu1036Cysfs*108	Exon18	.	[3]

Small in-frame deletions	c.3135del	p.Phe1046Serfs*3	Exon18	.	[3]
Small in-frame deletions	c.3139del	p.Leu1047Phefs*2	Exon18	.	[3]
Small in-frame deletions	c.3139_3142del	p.Leu1047*	Exon18	.	[3]
Small in-frame deletions	c.3146A>T	p.Asn1049Ile	.	.	[3]
Small in-frame deletions	c.3150del	p.Trp1051Glyfs*7	Exon18	.	[3]
Small in-frame deletions	c.3181_3186del	p.Ala1061_Leu1062del	Exon19	Germline	[28]
Small in-frame deletions	c.3233_3239del	p.Leu1078Profs*7	Exon19	.	[3]
Small in-frame deletions	c.3244_3249del6	p.Pro1082_Val1083del	Exon19	Germline	[16]
Small in-frame deletions	c.3251_3272del	p.Val1084Alafs*2	Exon19	.	[3]
Small in-frame deletions	c.3280del	p.Val1094Trpfs*12	.	.	[30]
Small in-frame deletions	c.3364_3365del	p.Met1122Valfs*22	Exon20	.	[3]
Small in-frame deletions	c.3371del	p.Ala1124Aspfs*15	.	.	[30]
Small in-frame deletions	c.3375del	p.Val1126Serfs*13	Exon20	.	[3]
Small in-frame deletions	c.3394T>C	p.Ser1132Pro	.	.	[24]
Small in-frame deletions	c.3475del	p.Leu1159Serfs*32	Exon21	.	[58]
Small in-frame deletions	c.3525_3526del	p.Leu1175Phefs*8	Exon21	.	[3]
Small in-frame deletions	c.3531_3534del	p.Phe1177Leufs*13	.	.	[64]
Small in-frame deletions	c.4048C>T	p.Arg1350Trp	Exon23	.	[3]
Small out-of-frame insertions	c.264_265insAATA	p.Lys89Asnfs*52	Exon2	Somatic	[53]
Small out-of-frame insertions	c.282_283insA	p.Gln95Thrfs*45	Exon2	Germline	[34]
Small out-of-frame insertions	c.323_324insGA	p.Ile108Metfs*10	Exon2	Germline	[12]
Small out-of-frame insertions	c.361_362insGAGC	p.Leu121Argfs*20	Exon2	Somatic	[1]
Small in-frame insertions	c.1338_1339insGCG	p.Tyr446_Leu447insAla	Exon9	Germline	[1][46]
Small in-frame insertions	c.2178_2179insA	p.Cys727Metfs*11	Exon14	.	[3]
Small duplications	c.280dup	p.Ile94Asnfs*46	Exon2	Germline	[13]
Small duplications	c.290dupA	p.Asn97Lysfs*43	Exon2	Germline	[65]
Small duplications	c.385_386dup	p.Trp129Cysfs*9	Exon2	Germline	[3]
Small duplications	c.572_575dup	p.Met192Ilefs*61	Exon3	Germline	[3]
Small duplications	c.705dupC	p.Trp236Leufs*16	Exon5	Germline	[27]
Small duplications	c.919dup	p.Thr307Asnfs*12	.	.	[30]
Small duplications	c.931dupA	p.Asn312Lysfs*7	Exon6	Germline	[18][19]
Small duplications	c.935dup	p.Asn312Lysfs*7	.	.	[12]
Small duplications	c.955dup	p.Met319Asnfs*118	.	.	[30]
Small duplications	c.1027_1028dupGT	p.Gly344Thrfs*24	Exon7	Germline	[4]
Small duplications	c.1028_1029dup	p.Gly344Trpfs*24	.	.	[4]
Small duplications	c.1043_1046dupAGAA	p.Asn349Lysfs*88	Exon7	Germline	[66]
Small duplications	c.1100_1108dup	p.Met367_Pro369dup	.	.	[30]
Small duplications	c.1138_1195dup58	p.Trp399*	Exon8	Germline	[43]

Small duplications	c.1259dupT	p.Ser421Phefs*16	Exon9	Germline	[67]
Small duplications	c.1261dupT	p.Ser421Phefs*16	Exon9	Germline	[17]
Small duplications	c.1349dupT	p.Ala451Argfs*46	Exon10	Germline	[68]
Small duplications	c.1366dup	p.Thr456Asnfs*41	Exon10	.	[3]
Small duplications	c.1370dupT	p.Met457Ilefs*40	Exon10	Germline	[69]
Small duplications	c.1353_1377dup	p.Trp460Leufs*45	.	.	[70]
Small duplications	c.1467dupA	p.Leu490Ilefs*7	Exon10	Germline	[71]
Small duplications	c.1508dup	p.Leu503Phefs*12	Exon11	.	[3]
Small duplications	c.1651dupA	p.Thr551Asnfs*76	Exon12	Germline	[34]
Small duplications	c.1723dupC	p.Leu575Profs*52	Exon12	Germline	[34]
Small duplications	c.1925dup	p.Pro643Thrfs*11	Exon14	.	[3]
Small duplications	c.2011dup	p.His671Profs*10	Exon14	.	[3]
Small duplications	c.2011_2012dupCA	p.His673Argfs*21	Exon14	Germline	[12]
Small duplications	c.2178dupC	p.Cys727Leufs*17	Exon14	Germline	[13]
Small duplications	c.2256_2262dup7	p.Phe755Glyfs*37	Exon15	Germline	[72]
Small duplications	c.2392dupC	p.Phe798Leufs*31	Exon15	Germline	[71]
Small duplications	c.2454dupA	p.Leu819Thrfs*10	Exon15	Germline	[2]
Small duplications	c.2535dupG	p.His846Argfs*15	Exon15	Germline	[73]
Small duplications	c.2712dupA	p.Gln905Thrfs*11	Exon17	Germline	[47]
Small duplications	c.2795_2799dup5	p.Tyr934Serfs*30	Exon17	Germline	[16]
Small duplications	c.2918_2925dup8	p.Phe976Serfs*22	Exon18	Germline	[11]
Small duplications	c.2960_2961dup	p.Val988Leufs*8	.	.	[49]
Small duplications	c.3025dup	p.Tyr1009Leufs*136	.	.	[74]
Small duplications	c.3026dupA	p.Tyr1009*	Exon18	Germline	[27]
Small duplications	c.3244_3246dupCCC	p.Pro1082dup	Exon19	Germline	[6]
Small duplications	c.3325_3328dupGGCG	p.Asp1110Glyfs*36	Exon20	Germline	[75]
Small duplications	c.3497dup	p.Asn1166Lysfs*18	Exon21	.	[3]

Reference

1. Li, T.J.; Yuan, J.W.; Gu, X.M.; Sun, L.S.; Zhao, H.S. PTCH germline mutations in Chinese nevoid basal cell carcinoma syndrome patients. *Oral Dis.* **2008**, *14*, 174–179, doi:10.1111/j.1601-0825.2007.01369.x.
2. Tanioka, M.; Takahashi, K.; Kawabata, T.; Kosugi, S.; Murakami, K.I.; Miyachi, Y.; Nishigori, C.; Iizuka, T. Germline mutations of the PTCH gene in Japanese patients with nevoid basal cell carcinoma syndrome. *Arch. Dermatol. Res.* **2005**, *296*, 303–308, doi:10.1007/s00403-004-0520-1.
3. Reinders, M.G.; van Hout, A.F.; Cosgun, B.; Paulussen, A.D.; Leter, E.M.; Steijlen, P.M.; Mosterd, K.; van Geel, M.; Gille, J.J. New mutations and an updated database for the patched-1 (PTCH1) gene. *Mol. Genet. Genomic Med.* **2018**, *6*, 409–415, doi:10.1002/mgg3.380.
4. Savino, M.; d'Apolito, M.; Formica, V.; Baorda, F.; Mari, F.; Renieri, A.; Carabba, E.; Tarantino, E.; Andreucci, E.; Belli, S.; et al. Spectrum of PTCH mutations in Italian nevoid basal cell-carcinoma syndrome patients: identification of thirteen novel alleles. *Hum. Mutat.* **2004**, *24*, 441, doi:10.1002/humu.9289.
5. Zhang, T.; Chen, M.; Lü, Y.; Xing, Q.; Chen, W. A novel mutation of the PTCH1 gene activates the Shh/Gli signaling pathway in a Chinese family with nevoid basal cell carcinoma syndrome. *Biochem. Biophys. Res.*

Commun. **2011**, *409*, 166–170, doi:10.1016/j.bbrc.2011.04.047.

6. Sun, L.S.; Li, X.F.; Li, T.J. PTCH1 and SMO gene alterations in keratocystic odontogenic tumors. *J. Dent. Res.* **2008**, *87*, 575–579, doi:10.1177/154405910808700616.
7. Alonso-González, J.; Gutiérrez-González, E.; Fernández-Redondo, V.; Vega-Gliemmo, A.; Toribio, J. Variable expression of naevoid basal cell carcinoma syndrome in a family with a novel mutation in the PTCH1 gene. *Clin. Exp. Dermatol.* 2012, *37*, 311–313.
8. Ogata, K.; Ikeda, M.; Miyoshi, K.; Yamamoto, Y.; Yamamoto, T.; Osaki, T.; Michimukai, E.; Tanaka, Y.; Sakamoto, A.; Oakamoto, T.; et al. Naevoid basal cell carcinoma syndrome with a palmar epidermoid cyst, milia and maxillary cysts [3]. *Br. J. Dermatol.* 2001, *145*, 508–509.
9. Nakamura, M.; Tokura, Y. A novel missense mutation in the PTCH1 gene in a premature case of nevoid basal cell carcinoma syndrome. *Eur. J. Dermatology* 2009, *19*, 262–263.
10. Kitano, H.; Koyama, Y.; Komiya, M.; Sato, N.; Nakayama, T. Basal cell nevus syndrome: New mutation of the patched homologue 1 gene. *J. Oral Maxillofac. Surgery, Med. Pathol.* **2014**, *26*, 506–510, doi:10.1016/j.ajoms.2013.04.016.
11. Matsuzawa, N.; Nagao, T.; Shimozato, K.; Niikawa, N.; Yoshiura, K.I. Patched homologue 1 mutations in four Japanese families with basal cell nevus syndrome. *J. Clin. Pathol.* **2006**, *59*, 1084–1086, doi:10.1136/jcp.2005.029439.
12. Pastorino, L.; Cusano, R.; Nasti, S.; Faravelli, F.; Forzano, F.; Baldo, C.; Barile, M.; Gliori, S.; Muggianu, M.; Ghigliotti, G.; et al. Molecular characterization of Italian nevoid basal cell carcinoma syndrome patients. *Hum. Mutat.* **2005**, *25*, 322–323, doi:10.1002/humu.9317.
13. Boutet, N.; Bignon, Y.J.; Drouin-Garraud, V.; Sarda, P.; Longy, M.; Lacombe, D.; Gorry, P. Spectrum of PTCH1 mutations in French patients with Gorlin syndrome. *J. Invest. Dermatol.* **2003**, *121*, 478–481, doi:10.1046/j.1523-1747.2003.12423.x.
14. Onodera, S.; Saito, A.; Hasegawa, D.; Morita, N.; Watanabe, K.; Nomura, T.; Shibahara, T.; Ohba, S.; Yamaguchi, A.; Azuma, T. Multi-layered mutation in hedgehog-related genes in Gorlin syndrome may affect the phenotype. *PLoS One* **2017**, *12*, doi:10.1371/journal.pone.0184702.
15. Ng, D.; Stavrou, T.; Liu, L.; Taylor, M.D.; Gold, B.; Dean, M.; Kelley, M.J.; Dubovsky, E.C.; Vezina, G.; Nicholson, H.S.; et al. Retrospective family study of childhood medulloblastoma. *Am. J. Med. Genet.* **2005**, *134 A*, 399–403, doi:10.1002/ajmg.a.30653.
16. Chidambaram, A.; Goldstein, A.M.; Gailani, M.R.; Gerrard, B.; Bale, S.J.; DiGiovanna, J.J.; Bale, A.E.; Dean, M. Mutations in the Human Homologue of the Drosophila patched Gene in Caucasian and African-American Nevoid Basal Cell Carcinoma Syndrome Patients. *Cancer Res.* **1996**, *56*.
17. Fujii, K.; Kohno, Y.; Sugita, K.; Nakamura, M.; Moroi, Y.; Urabe, K.; Furue, M.; Yamada, M.; Miyashita, T. Mutations in the human homologue of Drosophila patched in Japanese nevoid basal cell carcinoma syndrome patients. *Hum. Mutat.* **2003**, *21*, 451–452, doi:10.1002/humu.9132.
18. Ponti, G.; Pastorino, L.; Pollio, A.; Nasti, S.; Pellacani, G.; Mignogna, M.D.; Tomasi, A.; Del Forno, C.; Longo, C.; Bianchi-Scarrà, G.; et al. Ameloblastoma: A neglected criterion for nevoid basal cell carcinoma (Gorlin) syndrome. *Fam. Cancer* **2012**, *11*, 411–418, doi:10.1007/s10689-012-9529-3.
19. Pastorino, L.; Pollio, A.; Pellacani, G.; Guarneri, C.; Ghiorzo, P.; Longo, C.; Bruno, W.; Giusti, F.; Bassoli, S.; Bianchi-Scarrà, G.; et al. Novel PTCH1 mutations in patients with keratocystic odontogenic tumors screened for nevoid basal cell carcinoma (NBCC) syndrome. *PLoS One* **2012**, *7*, doi:10.1371/journal.pone.0043827.
20. Ponti, G.; Pollio, A.; Mignogna, M.D.; Pellacani, G.; Pastorino, L.; Bianchi-Scarrà, G.; Di Gregorio, C.; Magnoni,

C.; Azzoni, P.; Greco, M.; et al. Unicystic ameloblastoma associated with the novel K729M PTCH1 mutation in a patient with nevoid basal cell carcinoma (Gorlin) syndrome. *Cancer Genet.* **2012**, *205*, 177–181, doi:10.1016/j.cancergen.2012.01.012.
21. Torrelo, A.; Hernández-Martín, A.; Bueno, E.; Colmenero, I.; Rivera, I.; Requena, L.; Happle, R.; González-Sarmiento, R. Molecular evidence of type 2 mosaicism in Gorlin syndrome. *Br. J. Dermatol.* **2013**, *169*, 1342–1345, doi:10.1111/bjd.12458.
22. Li, J.; Wang, J.; Liu, Y.; Wang, W. Analysis of mutation in exon 17 of PTCH in patients with nevoid basal cell carcinoma syndrome. *Mol. Biol. Rep.* **2010**, *37*, 359–362, doi:10.1007/s11033-009-9782-1.
23. Ponti, G.; Pollio, A.; Pastorino, L.; Pellacani, G.; Magnoni, C.; Nasti, S.; Fortuna, G.; Tomasi, A.; Scarrà, G.B.; Seidenari, S. Patched homolog 1 gene mutation (p.G1093R) induces nevoid basal cell carcinoma syndrome and non-syndromic keratocystic odontogenic tumors: A case report. *Oncol. Lett.* **2012**, *4*, 241–244, doi:10.3892/ol.2012.707.
24. Reifenberger, J.; Arnold, N.; Kiechle, M.; Reifenberger, G.; Hauschild, A. Coincident PTCH and BRCA1 germline mutations in a patient with nevoid basal cell carcinoma syndrome and familial breast cancer [2]. *J. Invest. Dermatol.* 2001, *116*, 472–474.
25. Otsubo, S.; Honma, M.; Asano, K.; Takahashi, H.; Iizuka, H. A novel germ-line mutation of PTCH1 gene in a Japanese family of nevoid basal cell carcinoma syndrome: Are the palmoplantar pits associated with true basal cell carcinoma? *J. Dermatol. Sci.* 2008, *51*, 144–146.
26. Barreto, D.C.; Gomez, R.S.; Bale, A.E.; Boson, W.L.; De Marco, L. PTCH gene mutations in odontogenic keratocysts. *J. Dent. Res.* **2000**, *79*, 1418–1422, doi:10.1177/00220345000790061101.
27. Lench, N.J.; Telford, E.A.R.; High, A.S.; Markham, A.F.; Wicking, C.; Wainwright, B.J. Characterisation of human patched germ line mutations in naevoid basal cell carcinoma syndrome. *Hum. Genet.* **1997**, *100*, 497–502, doi:10.1007/s004390050541.
28. Guo, Y.Y.; Zhang, J.Y.; Li, X.F.; Luo, H.Y.; Chen, F.; Li, T.J. PTCH1 Gene Mutations in Keratocystic Odontogenic Tumors: A Study of 43 Chinese Patients and a Systematic Review. *PLoS One* **2013**, *8*, doi:10.1371/journal.pone.0077305.
29. Minami, M.; Urano, Y.; Ishigami, T.; Tsuda, H.; Kusaka, J.; Arase, S. Germline mutations of the PTCH gene in Japanese patients with nevoid basal cell carcinoma syndrome. *J. Dermatol. Sci.* **2001**, *27*, 21–26, doi:10.1016/S0923-1811(01)00104-9.
30. Klein, R.D.; Dykas, D.J.; Bale, A.E. Clinical testing for the nevoid basal cell carcinoma syndrome in a DNA diagnostic laboratory. *Genet. Med.* **2005**, *7*, 611–619, doi:10.1097/01.gim.0000182879.57182.b4.
31. Suzuki, M.; Hatsuse, H.; Nagao, K.; Takayama, Y.; Kameyama, K.; Kabasawa, Y.; Omura, K.; Yoshida, M.; Fujii, K.; Miyashita, T. Selective haploinsufficiency of longer isoforms of PTCH1 protein can cause nevoid basal cell carcinoma syndrome. *J. Hum. Genet.* **2012**, *57*, 422–426, doi:10.1038/jhg.2012.45.
32. Pan, S.; Li, T.J. PTCH1 mutations in odontogenic keratocysts: Are they related to epithelial cell proliferation? *Oral Oncol.* **2009**, *45*, 861–865, doi:10.1016/j.oraloncology.2009.02.003.
33. Pan, S.; Xu, L. li; Sun, L. sha; Li, T. jun Identification of known and novel PTCH mutations in both syndromic and non-syndromic keratocystic odontogenic tumors. *Int. J. Oral Sci.* **2009**, *1*, 34–38, doi:10.4248/ijos.08013.
34. Wicking, C.; Gillies, S.; Smyth, I.; Shanley, S.; Fowles, L.; Ratcliffe, J.; Wainwright, B.; Chenevix-Trench, G. De novo mutations of the patched gene in nevoid basal cell carcinoma syndrome help to define the clinical phenotype. *Am. J. Med. Genet.* **1997**, *73*, 304–307, doi:10.1002/(SICI)1096-8628(19971219)73:3<304::AID-AJMG14>3.0.CO;2-N.

35. Song, Y.L.; Zhang, W.F.; Peng, B.; Wang, C.N.; Wang, Q.; Bian, Z. Germline mutations of the PTCH gene in families with odontogenic keratocysts and nevoid basal cell carcinoma syndrome. *Tumor Biol.* **2006**, *27*, 175–180, doi:10.1159/000093054.
36. Endo, M.; Fujii, K.; Miyashita, T.; Uchikawa, H.; Tanabe, R.; Sugita, K.; Arai, H.; Kohno, Y. PTCH1 gene analysis in 25 Japanese patients with Gorlin syndrome. *No To Hattatsu* **2009**, *41*, 259–263, doi:10.11251/ojjscn.41.259.
37. Shevchenko, Y.O.; Bale, S.J.; Compton, J.G. Mutation Screening Using Automated Bidirectional Dideoxy Fingerprinting. *Biotechniques* **2000**, *28*, 134–138, doi:10.2144/00281rr01.
38. K, H.-T.; V, B.; J, L.; F, O.; JR, S.; AM, F. Gorlin syndrome: identification of 4 novel germ-line mutations of the human patched (PTCH) gene. Mutations in brief no. 137. Online. *Hum. Mutat.* **1998**, *11*, doi:10.1002/(SICI)1098-1004(1998)11:6<480::AID-HUMU9>3.0.CO;2-4.
39. Romano, M.; Iacovello, D.; Cascone, N.C.; Contestabile, M.T. Identification of a novel mutation in the PTCH gene in a patient with Gorlin-Goltz syndrome with unusual ocular disorders. *Eur. J. Ophthalmol.* **2011**, *21*, 516–519, doi:10.5301/EJO.2010.6186.
40. Hahn, H.; Wicking, C.; Zaphiropoulos, P.G.; Gailani, M.R.; Shanley, S.; Chidambaram, A.; Vorechovsky, I.; Holmberg, E.; Unden, A.B.; Gillies, S.; et al. Mutations of the human homolog of drosophila patched in the nevoid basal cell carcinoma syndrome. *Cell* **1996**, *85*, 841–851, doi:10.1016/S0092-8674(00)81268-4.
41. Scott, A.; Strouthidis, N.G.; Robson, A.G.; Forsyth, J.; Maher, E.R.; Schlottmann, P.G.; Michaelides, M. Bilateral Epiretinal Membranes in Gorlin Syndrome Associated With a Novel PTCH Mutation. *Am. J. Ophthalmol.* **2007**, *143*, 346–348, doi:10.1016/j.ajo.2006.09.022.
42. Wicking, C.; Shanley, S.; Smyth, I.; Gillies, S.; Negus, K.; Graham, S.; Suthers, G.; Haites, N.; Edwards, M.; Wainwright, B.; et al. Most germ-line mutations in the nevoid basal cell carcinoma syndrome lead to a premature termination of the patched protein, and no genotype- phenotype correlations are evident. *Am. J. Hum. Genet.* **1997**, *60*, 21–26.
43. Takahashi, C.; Kanazawa, N.; Yoshikawa, Y.; Yoshikawa, R.; Saitoh, Y.; Chiyo, H.; Tanizawa, T.; Hashimoto-Tamaoki, T.; Nakano, Y. Germline PTCH1 mutations in Japanese basal cell nevus syndrome patients. *J. Hum. Genet.* **2009**, *54*, 403–408, doi:10.1038/jhg.2009.55.
44. Reinders, M.G.H.C.; Boersma, H.J.; Leter, E.M.; Vreeburg, M.; Paulussen, A.D.C.; Arits, A.H.M.M.; Roemen, G.M.J.M.; Speel, E.J.M.; Steijlen, P.M.; van Geel, M.; et al. Postzygotic mosaicism in basal cell naevus syndrome. *Br. J. Dermatol.* **2017**, *177*, 249–252, doi:10.1111/bjd.15082.
45. Kansal, A.; Brueton, L.; Lahiri, A.; Lester, R. Hypoplastic thumb in Gorlin's syndrome. *J. Plast. Reconstr. Aesthetic Surg.* **2007**, *60*, 440–442, doi:10.1016/j.bjps.2006.06.006.
46. Gu, X.M.; Zhao, H.S.; Sun, L.S.; Li, T.J. PTCH mutations in sporadic and Gorlin-syndrome-related odontogenic keratocysts. *J. Dent. Res.* **2006**, *85*, 859–863, doi:10.1177/154405910608500916.
47. Fujii, M.; Noguchi, K.; Urade, M.; Muraki, Y.; Moridera, K.; Kishimoto, H.; Hashimoto-Tamaoki, T.; Nakano, Y. Novel PTCH1 mutations in Japanese Nevoid basal cell carcinoma syndrome patients: Two familial and three sporadic cases including the first Japanese patient with medulloblastoma. *J. Hum. Genet.* **2011**, *56*, 277–283, doi:10.1038/jhg.2011.2.
48. Smyth, I.; Wicking, C.; Wainwright, B.; Chenevix-Trench, G. The effects of splice site mutations in patients with naevoid basal cell carcinoma syndrome. *Hum. Genet.* **1998**, *102*, 598–601, doi:10.1007/s004390050747.
49. Soufir, N.; Gerard, B.; Portela, M.; Brice, A.; Liboutet, M.; Saiag, P.; Descamps, V.; Kerob, D.; Wolkenstein, P.; Gorin, I.; et al. PTCH mutations and deletions in patients with typical nevoid basal cell carcinoma syndrome

and in patients with a suspected genetic predisposition to basal cell carcinoma: A French study. *Br. J. Cancer* **2006**, *95*, 548–553, doi:10.1038/sj.bjc.6603303.

50. Pruvost-Balland, C.; Gorry, P.; Boutet, N.; Magnaldo, T.; Mamelle, G.; Margulis, A.; Kolb, F.; Duvillard, P.; Spatz, A.; Brugières, L.; et al. Étude clinique et recherche de mutations germinales du gène PTCH 1 dans le syndrome des hamartomes basocellulaires. *Ann. Dermatol. Venereol.* **2006**, *133*, 117–123, doi:10.1016/S0151-9638(06)70861-4.
51. Wilson, L.C.; Ajayi-Obe, E.; Bernhard, B.; Maas, S.M. Patched mutations and hairy skin patches: A new sign in Gorlin syndrome. In Proceedings of the American Journal of Medical Genetics, Part A; Am J Med Genet A, 2006; Vol. 140, pp. 2625–2630.
52. Abe, S.; Kabashima, K.; Sakabe, J.I.; Shimauchi, T.; Van, Z.; Okamoto, T.; Tokura, Y. Coincident two mutations and one single nucleotide polymorphism of the PTCH1 gene in a family with naevoid basal cell carcinoma syndrome. *Acta Derm. Venereol.* 2008, *88*, 635–636.
53. Sasaki, R.; Miyashita, T.; Matsumoto, N.; Fujii, K.; Saito, K.; Ando, T. Multiple keratocystic odontogenic tumors associated with nevoid basal cell carcinoma syndrome having distinct PTCH1 mutations: A case report. *Oral Surgery, Oral Med. Oral Pathol. Oral Radiol. Endodontology* **2010**, *110*, doi:10.1016/j.tripleo.2010.04.006.
54. Kadlub, N.; Coudert, A.; Gatibelza, M.E.; El Houmami, N.; Soufir, N.; Ruhin-Poncet, B.; L'Hermine, A.C.; Berdal, A.; Vazquez, M.P.; Descroix, V.; et al. PTCH1 mutation and local aggressiveness of odontogenic keratocystic tumors in children: Is there a relationship? *Hum. Pathol.* **2013**, *44*, 1071–1078, doi:10.1016/j.humpath.2012.09.012.
55. Johnson, R.L.; Rothman, A.L.; Xie, J.; Goodrich, L. V.; Bare, J.W.; Bonifas, J.M.; Quinn, A.G.; Myers, R.M.; Cox, D.R.; Epstein, E.H.; et al. Human homolog of patched, a candidate gene for the basal cell nevus syndrome. *Science (80-.).* **1996**, *272*, 1668–1671, doi:10.1126/science.272.5268.1668.
56. Seracchioli, R.; Bagnoli, A.; Colombo, F.M.; Missiroli, S.; Venturoli, S. Conservative treatment of recurrent ovarian fibromas in a young patient affected by Gorlin syndrome.
57. García De Marcos, J.A.; Dean-Ferrer, A.; Arroyo Rodríguez, S.; Calderón-Polanco, J.; Alamillos Granados, F.J.; Poblet, E. Basal cell nevus syndrome: Clinical and genetic diagnosis. *Oral Maxillofac. Surg.* **2009**, *13*, 225–230, doi:10.1007/s10006-009-0169-1.
58. Kitsiou-Tzeli, S.; Willems, P.; Kosmadaki, M.; Leze, E.; Vrettou, C.; Kanavakis, E.; Katsarou, A. Nevoid basal carcinoma syndrome (Gorlin syndrome) and pronounced androgenic alopecia in a woman with a novel mutation p.Leu1159fsx32 in the PTCH gene. *J. Dermatol.* 2011, *38*, 1205–1208.
59. Detecting tissue-specific alternative splicing and disease-associated aberrant splicing of the PTCH gene with exon junction microarrays | Human Molecular Genetics | Oxford Academic.
60. Škodrić-Trifunović, V.; Stjepanović, M.; Savić, Ž.; Ilić, M.; Kavečan, I.; Privrodski, J.J.; Spasovski, V.; Stojiljković, M.; Pavlović, S. Novel Patched 1 mutations inpatients with nevoid basal cellcarcinoma syndrome - case report. *Croat. Med. J.* **2015**, *56*, 63–67, doi:10.3325/cmj.2015.56.63.
61. Lü, Y.; Zhu, H.; Ye, W.; Zhang, M.; He, D.; Chen, W. A new mutation of PTCH gene in a Chinese family with nevoid basal cell carcinoma syndrome. *Chin. Med. J. (Engl).* **2008**, *121*, 118–21.
62. Le Brun Keris, Y.; Jouk, P.S.; Saada-Sebag, G.; Roux, J.J.; Mattei, B.; Bagait, L.; Paoloni-Giacobino, A.; Grandchamp, B.; Soufir, N.; Lespinasse, J. Prenatal manifestation in a family affected by nevoid basal cell carcinoma syndrome. *Eur. J. Med. Genet.* **2008**, *51*, 472–478, doi:10.1016/j.ejmg.2008.04.002.
63. Jimbo, T.; Masumoto, K.; Urita, Y.; Takayasu, H.; Shinkai, T.; Uesugi, T.; Gotoh, C.; Sakamoto, N.; Sasaki, T.; Oto, T.; et al. Nevoid basal cell carcinoma syndrome with a unilateral giant ovarian fibroma in a Japanese 6-

year-old girl. *Eur. J. Pediatr.* **2014**, *173*, 667–670, doi:10.1007/s00431-013-2200-7.

64. Marsh, A.; Wicking, C.; Wainwright, B.; Chenevix-Trench, G. DHPLC analysis of patients with Nevoid Basal Cell Carcinoma Syndrome reveals novel PTCH missense mutations in the sterol-sensing domain. *Hum. Mutat.* **2005**, *26*, 283, doi:10.1002/humu.9365.
65. Kijima, C.; Miyashita, T.; Suzuki, M.; Oka, H.; Fujii, K. Two cases of nevoid basal cell carcinoma syndrome associated with meningioma caused by a PTCH1 or SUFU germline mutation. *Fam. Cancer* **2012**, *11*, 565–570, doi:10.1007/s10689-012-9548-0.
66. Musani, V.; Gorry, P.; Basta-Juzbasic, A.; Stipic, T.; Miklic, P.; Levanat, S. Mutation in exon 7 of PTCH deregulates SHH/PTCH/SMO signaling: Possible linkage to WNT. *Int. J. Mol. Med.* **2006**, *17*, 755–759, doi:10.3892/ijmm.17.5.755.
67. Fujii, K.; Miyashita, T.; Omata, T.; Kobayashi, K.; Takanashi, J.I.; Kouchi, K.; Yamada, M.; Kohno, Y. Gorlin syndrome with ulcerative colitis in a Japanese girl. *Am. J. Med. Genet.* **2003**, *121 A*, 65–68, doi:10.1002/ajmg.a.20082.
68. Geneviève, D.; Walter, E.; Gorry, P.; Jacquemont, M.L.; Dupic, L.; Layet, V.; Munnich, A.; Cormier-Daire, V.; Dommergues, M.; Lyonnet, S.; et al. Gorlin syndrome presenting as prenatal chylothorax in a girl. *Prenat. Diagn.* **2005**, *25*, 997–999, doi:10.1002/pd.1231.
69. Tostar, U.; Malm, C.J.; Meis-Kindblom, J.M.; Kindblom, L.G.; Toftgård, R.; Undén, A.B. Deregulation of the hedgehog signalling pathway: A possible role for the PTCH and SUFU genes in human rhabdomyoma and rhabdomyosarcoma development. *J. Pathol.* **2006**, *208*, 17–25, doi:10.1002/path.1882.
70. Mazzuoccolo, L.D.; Martínez, M.F.; Muchnik, C.; Azurmendi, P.J.; Stengel, F. SÍNDROME DE CARCINOMA BASOCELULAR NEVOIDE Y AGENESIA DE CUERPO CALLOSO SÍNDROME DE CARCINOMA BASOCELULAR NEVOIDE CON AGENESIA DE CUERPO CALLOSO, MUTACIÓN EN PTCH1 Y AUSENCIA DE CARCINOMA BASOCELULAR. *CASUÍSTICA Med. (Buenos Aires)* **2014**, *74*, 307–310.
71. Lam, C.W.; Leung, C.Y.; Lee, K.C.; Xie, J.; Lo, F.M.; Au, T.S.; Tong, S.F.; Poon, M.K.; Chan, L.Y.; Luk, N.M. Novel mutations in the PATCHED gene in basal cell nevus syndrome. *Mol. Genet. Metab.* **2002**, *76*, 57–61, doi:10.1016/S1096-7192(02)00021-5.
72. Muramatsu, S.; Suga, Y.; Mizuno, Y.; Hasegawa, T.; Komuro, Y.; Kubo, Y.; Imakado, S.; Ogawa, H. A Japanese case of naevoid basal cell carcinoma syndrome associated with segmental vitiligo [8]. *Br. J. Dermatol.* 2005, *152*, 812–814.
73. Pastorino, L.; Cusano, R.; Baldo, C.; Forzano, F.; Nasti, S.; Di Rocco, M.; Carta, M.; Dagna Bricarelli, F.; Faravelli, F.; Bianchi Scarrà, G. Nevoid Basal Cell Carcinoma Syndrome in infants: Improving diagnosis. *Child. Care. Health Dev.* **2005**, *31*, 351–354, doi:10.1111/j.1365-2214.2005.00514.x.
74. García-Oguiza, A.; Miralbés-Terraza, S.; Calvo-Martín, M.T.; Labarta-Aizpun, J.; López-Pisón, J.; Marco-Tello, A.; Rebage Moisés, V. Neonatal Gorlin syndrome associated to hemimegalencephaly confirmed by genetic study [1]. *Rev. Neurol.* 2006, *43*, 251–253.
75. Sasaki, R.; Saito, K.; Watanabe, Y.; Takayama, Y.; Fujii, K.; Agawa, K.; Miyashita, T.; Ando, T.; Akizuki, T. Nevoid basal cell carcinoma syndrome with cleft lip and palate associated with the novel PTCH gene mutations. *J. Hum. Genet.* **2009**, *54*, 398–402, doi:10.1038/jhg.2009.51.
